# Radiation Myelopathy: A Case Report and Literature Review

**DOI:** 10.7759/cureus.41362

**Published:** 2023-07-04

**Authors:** Anika Iftekharuddin, Vadim Gospodarev, Namath S Hussain

**Affiliations:** 1 Neurological Surgery, Loma Linda University Medical Center, Loma Linda, USA

**Keywords:** delayed radiation myelopathy, radiation myelopathy, spinal cord lesion, radiation therapy, radiation oncology, metastatic disease

## Abstract

Proton beam therapy is a common type of radiation treatment that delivers a beam of proton particles to treat cancer and minimize damage to nearby healthy tissue. In this paper, we describe a case of a 20-year-old male patient with osteosarcoma of the distal right femur that eventually metastasized to his thoracic cavity. The patient underwent radiation beam therapy treatment that was directed at his left thorax and nine months later presented with clinical and radiographic findings of delayed radiation myelopathy (RM).

## Introduction

Radiation therapy (RT) utilizes ionizing radiation to destroy cancer cells. While it can be an effective form of therapy in treating many cancers, there is an inherent risk of affecting healthy tissue as well. The spinal cord is known to be particularly sensitive to radiation, which can result in radiation myelopathy (RM) [1]. RM is a rare complication of RT and is defined as damage to white matter in the spinal cord after exposure to ionizing radiation over a certain period of time [2,3]. Early injury RM is thought to occur after large dose radiation exposure to the central nervous system; however, large dose radiation to the spinal cord generally does not lead to acute symptoms. Subacute myelopathy is the most common type of RM and occurs after a latent period of four to six months after RT. The pathophysiology underlying subacute RM involves transient demyelination of the spinal cord. Since subacute myelopathy is self-limiting, it does not require treatment. Delayed RM (DRM) is a type of chronic, progressive RM. Unlike subacute myelopathy, DRM is irreversible and develops months to years after radiation exposure [4,5]. We hereby present a case report of a patient demonstrating DRM. Since DRM is a rare condition and can be a challenging diagnosis, the purpose of this paper is to present a case report and show one of the ways RM can manifest and contribute to the expanding literature about DRM.

## Case presentation

**Figure 1:**
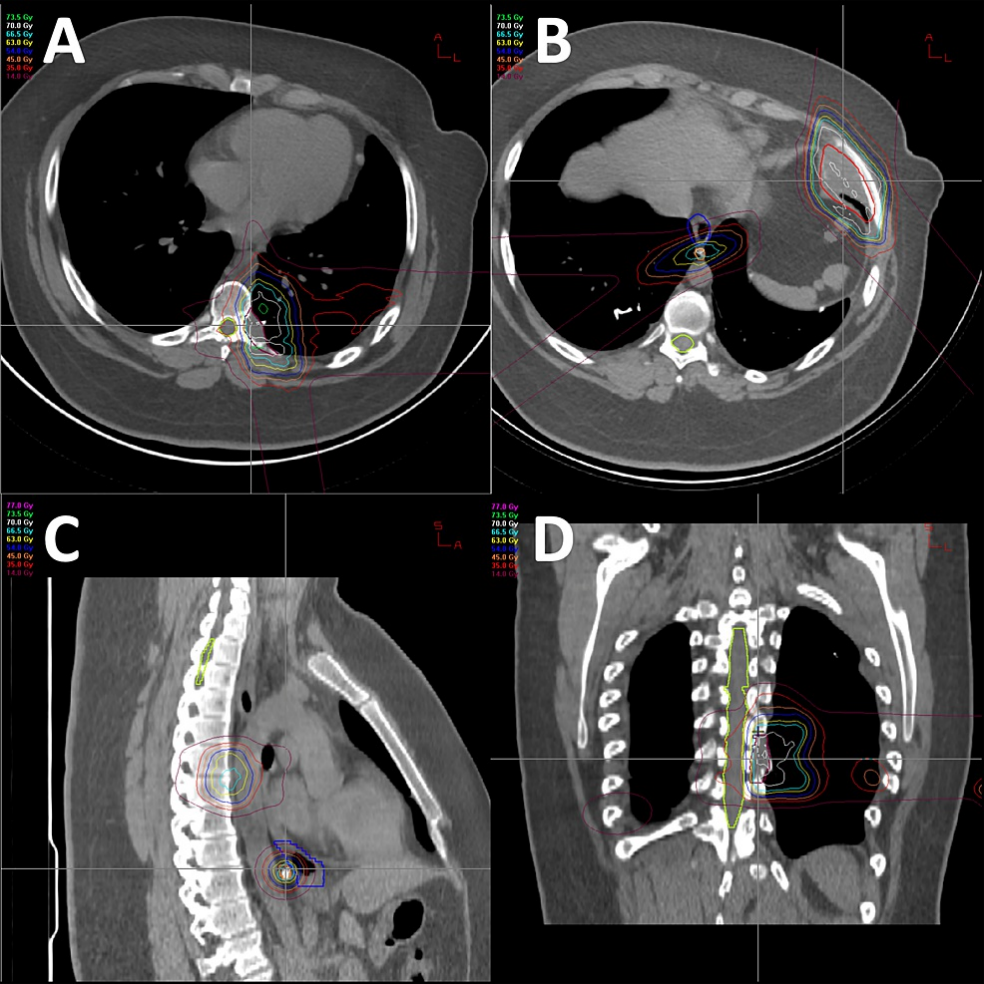
CT of the chest and radiation dose distributions A: CT of the chest axial view depicting radiation dose distributions targeting paraspinal lesion. B: CT of the chest axial view depicting radiation dose distributions targeting paraaortic and left anterior lower chest wall lesions. C: CT of the chest sagittal view depicting radiation dose distributions targeting paraaortic and paraspinal lesions. D: CT of the chest coronal view depicting radiation dose distributions targeting paraaortic, paraspinal, and mediastinal paraesophageal lesions

**Figure 2:**
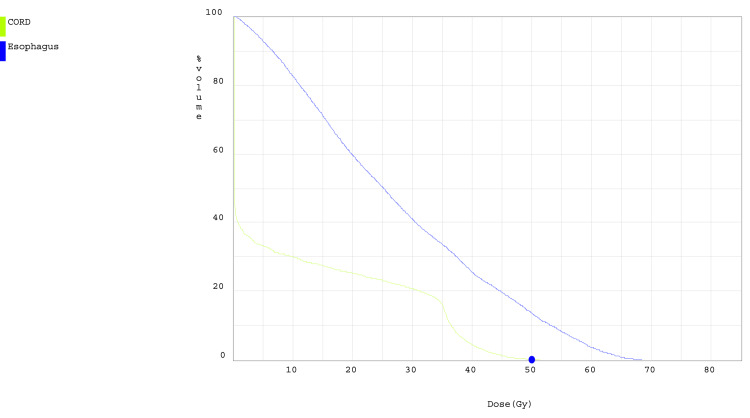
Spinal cord radiation exposure curve The spinal cord was exposed to a maximum total dose of 35 Gy based on the dose constraint and target volume curve depicted comparing exposure of the spinal cord (green) to the esophagus (blue)

**Figure 3:**
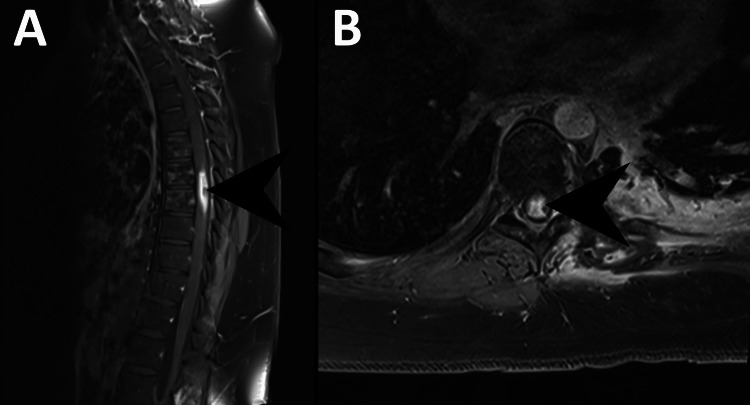
MRI A: Post-contrast MRI T1 sagittal view of the thoracic spine, demonstrating cord enhancement extending from T7-T8, most suggestive of radiation necrosis (black arrowhead). B: Post-contrast MRI T1 axial view of the thoracic spine, re-demonstrating cord enhancement (black arrowhead)

The patient is a 20-year-old male who was initially diagnosed with osteosarcoma of the distal right femur and underwent radical resection of the lesion at the age of 14. Since the time of his initial diagnosis, the patient was enrolled in the AOST0331 clinical trial. Three years after his initial diagnosis, he was found to have evidence of thoracic metastatic disease with multiple pulmonary nodules that were suspicious for malignancy and underwent left upper lung lobe resection which confirmed metastatic high-grade osteosarcoma. At 19 years of age, the patient underwent proton beam therapy treatment directed at the left thorax for treatment of metastatic disease. The patient received a total of 70 Cobalt Gray equivalent of RT which was divided over a treatment course of five fractions per week with a 7 Gy dose per fraction for a total of 10 fractions. There were four lesions that the radiotherapy was directed against: paraspinal lesion (3.1 x 1.1 x 3.0 cm), paraaortic lesion (2.0 x 2.1 x 2.6 cm), left anterior lower chest wall lesion (4.1 x 2.6 x 3.7 cm), and a mediastinal paraesophageal lesion (0.8 x 1.7 x 0.8 cm). In order to deliver precise radiotherapy to targets of interest, the patient was immobilized in a full-body pod and a CT of the chest was obtained in order to plan dose distributions (Figure 1), which were then administered via daily stereoscopic X-ray image guidance technique. The spinal cord was exposed to a maximum total dose of 35 Gy based on the dose constraint and target volume curve depicted in Figure 2. Nine months after completion of proton beam therapy, the patient presented with left lower extremity numbness that was associated with weakness and impaired proprioception in the left lower extremity. The patient denied any recent trauma or falls and endorsed that the symptoms have been progressively getting worse over the course of two weeks. MRI of the lumbar and thoracic spine was performed, and although there were no abnormal findings in the lumbar spine, there was a 3.9 cm segment of enhancement noted to involve the left lateral aspect of the thoracic spinal cord extending from T7-8 (Figure 3).

The patient’s imaging findings, clinical presentation, and history of RT were concerning for DRM. Unfortunately, RM is an irreversible condition and treatment is usually supportive in nature. It was recommended that the patient start high-dose corticosteroids to reduce spinal cord edema and inflammation. The patient continued to have left lower extremity numbness associated with weakness and impaired proprioception during follow-up evaluation at nine months post-radiotherapy treatment, which has remained clinically stable.

## Discussion

Oligodendrocytes and endothelial cells are thought to be two types of cells affected in patients with RM [6]. RT uses ionizing radiation to generate free radicals that destroy DNA via double-stranded breaks, which results in chromosomal abnormalities and rearrangements [7]. Oligodendrocytes and multipotent stem cells are thought to be affected by RT, which leads to white matter changes [2]. When the axonal myelin sheath is damaged, neurons transmit action potentials at reduced speeds, which manifests as various neurological deficits [8]. Our patient's imaging revealed enhancement of the left lateral aspect of segments T7-T8, concerning demyelination in that segment, which explained his left lower extremity weakness, numbness, and loss of proprioception. RT can also affect astrocytes and microglial cells, which can produce cytokines such as vascular endothelial growth factor (VEGF). VEGF, in turn, is thought to affect endothelial cells and induce vascular hyperpermeability, which can lead to spinal cord edema [9]. Symptoms of DRM present months to years after RT exposure. Initial clinical manifestations include loss of temperature sensation and decreased proprioception. Over time, this can progress to ascending weakness, paresis and paralysis, hyperreflexia, and bowel and bladder sphincter dysfunction, among others. This process is irreversible, and the aforementioned neurological symptoms are very unlikely to remit [4,5,10]. Many other conditions such as infection, intramedullary neoplasms, rheumatic disease, and paraneoplastic syndromes can have similar manifestations as DRM; thus, these conditions must be ruled out before diagnosing patients with DRM. The diagnostic criteria of DRM include known spinal cord exposure to RT, neurological symptoms that correspond to the involved portion of the spinal cord [1,11], absence of primary neoplasm involving the spinal cord itself or cord compression by an external mass, and a minimum latency period of six months [12]. Our patient met much of the diagnostic criteria for DRM. He received RT to the left thorax for treatment of metastatic osteosarcoma affecting his lungs. The patient was treated with a total of 70 Gy that was administered in 10 fractions. The spinal cord was exposed to a maximum total dose of 35 Gy. Neurological examination of the patient demonstrated weakness, numbness, and loss of proprioception in the left lower extremity which corresponded to MRI findings that revealed enhancement involving the left lateral aspect of the thoracic spinal cord extending from T7-8 (Figure 3) and the patient's symptoms began approximately nine months after undergoing RT. Along with history and physical findings, MRI is frequently used to support the diagnosis of DRM. Affected portions of the spinal cord on MRI will demonstrate intramedullary T2 hyperintensity, T1 hypointensity, and enhancement with gadolinium T1-weighted images [13]. The enhancement is typically limited to the segment exposed to radiation and neighboring vertebral bodies may show fatty marrow change, which can further support the diagnosis of DRM [12]. MRI may demonstrate spinal cord edema, which is thought to be secondary to changes in vascular permeability due to endothelial cell exposure to RT [14,15]. Another type of imaging that can be useful in differentiating primary spinal cord lesions from metastatic disease to the spine is PET with fluorodeoxyglucose (FDG) which is a glucose analog. Metabolically active cells such as malignant tumors exhibit increased uptake of FDG, increased hexokinase activity, and expression of glucose transporters. Patients with RM have central nervous system cells that have been damaged by RT and will not exhibit high FDG uptake on PET. While there is no robust clinical data supporting RM prevention, some pre-clinical studies revealed that brief therapeutic intervention with platelet-derived growth factor and insulin-like growth factor-1 can possibly prevent DRM [16]. Animal studies have found that magnesium sulfate and vitamin E have neuroprotective effects and significantly reduce the amount of lipid peroxidation, a biochemical process thought to be involved in radiation-induced oxidative stress in the spinal cord [17]. X-ray radiation utilizes photons, and it is a highly penetrating form of ionizing RT. Unfortunately, much of the radiation traverses the target lesion and exits the body, which results in an exit dose. Proton beam therapy essentially eliminates the exit dose by utilizing charged particles whose depth can be controlled via calibration of beam energy and, thus, significantly reduces but does not completely eliminate the risk of causing damage to surrounding tissue [18]. Unfortunately, treatment options for DRM are limited and include glucocorticoids, antiangiogenic agents, hyperbaric oxygen, and physical therapy [19]. Glucocorticoids are most commonly used as the first-line treatment for suspected RM; however, steroid-induced myopathy that results from chronic use of steroids can worsen deficits due to RM. In small studies and case reports, the use of the antiangiogenic agent bevacizumab has resulted in the improvement of both radiographic findings and neurologic deficits. However, most of the improvement is seen in cranial radiographic findings rather than improvement in neurologic deficits [20,21]. Although the use of hyperbaric oxygen therapy is a recognized adjuvant treatment for osteoradionecrosis and radiation necrosis of soft tissues, its use in the treatment of RM is limited and is based upon anecdotal evidence derived from case reports [22,23]. Our patient was treated with high-dose corticosteroids, which did not improve or worsen his symptoms.

## Conclusions

DRM is a rare yet serious complication of RT. Some of the features that make DRM a challenging diagnosis include the vast array of neurological symptoms and the delay in the presentation of those symptoms that may take months to years after initial radiation exposure. The lack of effective measures to prevent or treat this condition and its irreversibility is especially concerning. It is, therefore, very important to inform patients of possible complications of RT before initiating treatment and monitor them closely for this condition. Should patients undergo treatment and develop clinical and radiographic evidence of DRM, clinicians should be well-equipped to know how to diagnose, manage and most importantly, educate their patients regarding this condition.
